# Successful Pregnancy in a 31-Year-Old Peritoneal Dialysis Patient with Bilateral Nephrectomy

**DOI:** 10.1155/2013/173405

**Published:** 2013-10-02

**Authors:** Ahmed Abu-Zaid, Ahmed Nazer, Osama AlOmar, Ismail A. Al-Badawi

**Affiliations:** ^1^College of Medicine, Alfaisal University, P.O. Box 50927, Riyadh 11533, Saudi Arabia; ^2^Department of Obstetrics and Gynecology, King Faisal Specialist Hospital and Research Center (KFSH & RC), P.O. Box 3354, Riyadh 11211, Saudi Arabia

## Abstract

Frequency of pregnancy among childbearing age women with end-stage renal disease (ESRD) undergoing long-term periodic dialysis ranges from 1% to 7%. Although pregnancy in dialysis women with ESRD is considered a largely high-risk pregnancy, occurrence of successful pregnancy is not impossible with success rates approaching 70%. Rates of successful pregnancy are greatly impacted by early pregnancy diagnosis and preserved residual renal functions. Herein, to the best of our knowledge, we report the first case of successful pregnancy (despite late diagnosis at 14 weeks of gestation) in a 31-year-old peritoneal dialysis patient with bilateral nephrectomy and no whatsoever preserved residual renal function. Moreover, a literature review on pregnancy in dialysis patients is presented.

## 1. Introduction

Pregnancy is largely uncommon among women with chronic kidney disease (CKD) and end-stage renal disease (ESRD) [1]. Incidence of conception among dialysis patients ranges from 1% to 7% [1], and this incidence appears to be higher among women with preserved residual renal functions [2, 3] and shorter intervals of long-term dialysis [4].

Pregnancy in women with chronic renal insufficiency possesses much higher maternal and fetal morbidity and mortality when compared to women with normal renal function [5, 6]. Although pregnancy in women with chronic renal insufficiency is considered a largely high-risk pregnancy, successful pregnancy for dialysis patients is not impossible [7]. Happily enough, with the existence of advanced dialysis systems and enhanced maternal and fetal care, the reported rate of successful pregnancies with delivery of surviving infants is reaching 70% [8–10]. The occurrence of successful pregnancy rate is hugely influenced by the early diagnosis of pregnancy, preserved residual renal function, prolonged dialysis, and appropriate multidisciplinary team management (obstetricians, nephrologists, dialysis nurses, and nutritionists) [7].

Herein, to the best of our knowledge, we report the first case of successful pregnancy (despite late diagnosis at 14 weeks of gestation) in a 31-year-old peritoneal dialysis woman with bilateral nephrectomy and no whatsoever preserved residual renal function. Moreover, a literature review on pregnancy in dialysis patients is presented.

## 2. Case Report

A 31-year-old Saudi woman, gravida 6 para 2, status postbilateral nephrectomy secondary to chronic pyelonephritis, pending renal transplantation, has been on regular peritoneal dialysis (18 hours/week) for 14 months, presented with amenorrhea for 3 months. Amenorrhea was associated with abdominal distention, nausea, and vomiting.

Blood laboratory investigations showed high betahuman chorionic gonadotropin (*β*-HCG) level (85,750 mIU/mL), which was highly suggestive of viable pregnancy. A pelvi-abdominal ultrasound confirmed a single 14-week gestational age fetus. Patient was referred to the maternal-fetal medicine and the nephrology departments for adequate counseling about maternal-fetal morbidity and mortality associated with pregnancy on peritoneal dialysis. The patient refused the termination of pregnancy and insistently elected to carry on with pregnancy. A multidisciplinary team involving obstetricians, gynecologists, nephrologists, dialysis nurses, and nutritionists was assigned to look after the patient. Peritoneal dialysis was increased to 22 hours/week.

At 24 weeks of gestation, the patient was admitted for close maternal and fetal surveillance. At admission, the mother had high blood pressure (169/109 mmHg), anemia (hemoglobin = 8.5 g/L), high blood urea nitrogen (BUN = 27 mg/dL), and few electrolytes disturbances and appeared malnourished.

As for the high blood pressure, mother was started on *α*-methyldopa 500 mg TID and nifedipine 60 mg BID. Blood pressure was not well controlled, and labetalol 200 mg BID was added. Blood pressure was well controlled afterwards with average readings of 126/93 mmHg. As for the anemia, mother received subcutaneous erythropoietin 8000 IU/week along with oral ferrous sulfate supplements 200 mg once daily. The mother did not require any blood transfusion, and the hemoglobin level was regularly maintained above 11.0 g/L throughout pregnancy. Ferritin saturation levels were frequently checked and persistently measured above 30%. As for the BUN, a targeted goal of predialysis BUN less than 50 mg/dL was set. The mother never exceeded 50 mg/dL and maintained a predialysis BUN average of 21 mg/dL throughout pregnancy. Peritoneal dialysis was adjusted to be daily (4 hours/day, 28 hours/week) using new biocompatible dialysis membranes with each dialysis. As for electrolyte disturbances, electrolytes (particularly calcium, potassium, and phosphorus) in blood and dialysate were adjusted accordingly. As for malnutrition, mother was started on 3000 calories and 100 grams of protein per day. Water-soluble vitamins were also supplemented as required. Vitamin D levels were within normal ranges and did not require supplements.

Fetal surveillance included serial ultrasound assessments, fetal heart rate monitoring, fetal nonstress test twice daily, and Doppler velocimetry measurements (umbilical artery and middle cerebral artery) once daily. Serial ultrasound assessments showed no polyhydramnios or other obstetric complications. Fetus was not appropriate for gestational age. Fetal heart rate was uniformly within normal ranges and fetal nonstress tests were regularly reactive. The average pulsatility indices ([PIs], systolic/diastolic ratio) for umbilical and middle cerebral arteries were 1.1 and 2.3 mmHg, respectively.

At 29 weeks of gestation, mother developed severe episodes of hypertension (despite the aggressive antihypertensive medications) along with severe proteinuria (more than +3). A diagnosis of preeclampsia was made. Ultrasound Doppler PIs for umbilical and middle cerebral arteries were 1.6 and 2.7 mmHg, respectively. An urgent Cesarean section was planned the next day, and, meanwhile, patient received subcutaneous administration of betamethasone phosphate (12 mg, 12 hours apart, for two doses) to promote fetal lung maturation.

Cesarean section was uneventful and delivered a preterm baby girl, weighed 780 g, with Apgar score of 5, 7, and 7 at 1, 5, and 10 minutes, respectively. Patient was transferred to the neonatal intensive care unit (NICU), intubated for 1 day, and received two doses of surfactant therapy. At day 60 postnatally, the baby girl weighed 1700 g, on nasal cannula, on full feeding, and discharged home without any adverse events of prematurity. The baby girl is still—up to this moment—thriving at 6 months of age with uneventful postnatal period. The mother is doing fine and still awaiting renal transplantation.

## 3. Discussion

Frequency of pregnancy among childbearing age women with advanced chronic kidney disease (CKD) and end-stage renal disease (ESRD) undergoing long-term periodic dialysis ranges from 1% to 7% [1]. However, this frequency of pregnancy tends to be much higher among women with preserved residual renal functions [2, 3] and shorter intervals of chronic dialysis (less than 10 years) [4].

Anemia and hyperprolactinemia largely contribute to the reduced fertility rate among women with chronic renal insufficiency [11]. Anemia correlates with poor health status and decreased libido. Conversely, hyperprolactinemia correlates with menstrual irregularities, amenorrhea, anovulation, depression, and reduced sexual function [8, 12]. Administration of erythropoietin has been shown to correct anemia, suppress prolactin levels, improve general health status, induce sexual drive, stimulate regular menstruation, and, ultimately, promote fertility [12, 13].

Diagnosing pregnancy in women who are undergoing dialysis is characteristically delayed [14, 15]. The average time at which a clinical diagnosis of pregnancy is established is roughly 16.5 weeks late [14]. This is because irregular menstruations and abdominal pain are already frequent symptoms in such individuals, and healthcare professionals are less likely to consider the diagnosis of pregnancy as a probable etiology of (for) these symptoms [15].

Urine pregnancy test for betahuman chorionic gonadotropin (*β*-HCG) is unhelpful in women with chronic renal insufficiency due to the distorted renal clearance [16]. Although blood pregnancy test for *β*-HCG is fairly reliable, abdominal sonography appears to be the most reliable method to confirm pregnancy and accordingly compute gestational age [16].

Early diagnosis of pregnancy is essential to offer the best possible antenatal, perinatal, and postpartum (postnatal) care by a multidisciplinary team involving obstetricians, nephrologists, dialysis nurses, and nutritionists [5]. Happily enough, with the existence of advanced dialysis systems and enhanced maternal and fetal care, the reported rate of successful pregnancies with the delivery of surviving infants is approximately 70% [8–10].

Infant mortality and low survival rates are coupled with particular obstetric adverse events occurring in dialysis patients throughout pregnancy. Such adverse events include premature rupture of membrane, preterm birth, polyhydramnios, intrauterine growth restriction, placental abruption, uncontrolled arterial hypertension, preeclampsia/eclampsia, hemorrhage, anemia, infection, and maternal demise [1, 17, 18].

Earlier studies have demonstrated that sufficient prolonged dialysis, appropriate hemodynamic steadiness (stability), proper management of obstetric complications, and adequate corrections of anemia and malnutrition are the most significant influencing factors for yielding uneventfully successful pregnancy in dialysis patients [7].

Increasing dialysis sessions/doses appears to improve pregnancy outcomes and provide various benefits [6, 10]. Extending dialysis time to 20 hours per week has been associated with higher baby birth weight, advanced gestational age, prolonged maternal-fetal life expectancy, and decreased risks of obstetric complications (such as polyhydramnios and preterm labor) [6]. Other two studies suggested that pregnant women with chronic renal failure should be subjected to the maximum possible time of dialysis on a daily basis and at least 24 hours per week [19, 20]. Other benefits of increasing dialysis sessions/doses include providing accurate measurement of estimated dry weight, supplying less toxic uremic environment for fetus, lowering predialysis blood urea nitrogen (BUN) levels, controlling hypertension and fluid intake, reducing the use of antihypertensive drugs, and correcting blood volume overloads, electrolytes disturbances, and acid-base imbalances [15].

The mode of dialysis remains debatable [7]. However, several studies revealed no significant differences in maternal and fetal outcomes among pregnant women on hemodialysis or peritoneal dialysis [1, 10, 21]. Moreover, many nephrologists and dialysis experts do not recommend changing the mode of dialysis after conception [7].

The use of new biocompatible dialysis membrane is highly recommended [17, 22] as it is associated with less teratogenicity [23] and reduced overall net protein catabolism [22] when compared to bioincompatible dialysis membrane. In the dialysate, a number of electrolyte levels should be adjusted accordingly. For example, potassium levels should be increased to 3–3.5 mmol/L to prevent hypokalemia [5, 6]. Conversely, bicarbonate levels should be maintained at low concentration levels (25 mEq/L) [1, 6]. This is because periodic dialysis has been shown to lead to increasingly inappropriate alkali shift to pregnant women, yielding alkalemia [6]. As 3.5 mEq/L dialysate calcium concentrations are associated with hypercalcemia, calcium levels in dialysate should be increased to a maximum of 2.5 mEq/L along with 1-2 g of calcium carbonate oral supplements [5, 6]. Since periodic dialysis can result in hypophosphatemia and adding phosphorous to dialysate can be challengingly problematic, oral phosphorous supplements and/or increased nutritional phosphorus ingestions are highly advised [24].

Maternal predialysis blood urea nitrogen (BUN) levels should be maintained below 50 mg/dL [5, 6, 12]. BUN levels below 50 mg/dL have been directly associated with more viable pregnancy, higher fetal birth weight, prolonged gestational age, and reduced hazards of obstetric complications, particularly polyhydramnios, premature rupture of membrane, and preterm birth [4, 21]. Fetal mortality straightforwardly correlates with BUN levels above 50 mg/dL, with hardly ever successful pregnancy rates happening in pregnant women with BUN values above 60 mg/dL in one study series [25].

Hypertension is the most commonly encountered maternal complication, affecting 42–80% of chronic dialysis pregnant women [2, 4]. This hypertension can be further complicated by proteinuria resulting into preeclampsia. If preeclampsia is not treated adequately, patient may develop eclampsia with life-threatening incidents of seizures during pregnancy. The mechanism of hypertension is not fully understood and is most likely multifactorial [26]. Although frequent dialysis can control fluid homeostasis and hypertension [10], some pregnant women develop uncontrolled arterial hypertension demanding the use of antihypertensive drugs [6]. The standard of care and most frequently used antihypertensive drugs are *α*-methyldopa, *β*-blockers (only labetalol), and hydralazine [4, 6]. In conditions of severe uncontrolled hypertension, calcium channel blockers (e.g., nifedipine, nicardipine, and verapamil), clonidine, and diuretics (e.g., furosemide) can be utilized safely with varying degrees of success [4, 6]. Angiotensin-converting enzyme inhibitors (ACEs) and angiotensin receptor blockers (ARBs) are contraindicated in pregnancy for their teratogenic effects (pregnancy category X) [5, 27]. As severely uncontrolled maternal hypertension often prompts urgent delivery, antihypertensive drugs can be used orally or intravenously in synergistic fashion with other drugs, in order to achieve the maximum possible optimal control of blood pressure.

Anemia is another frequently encountered complication in pregnant dialysis patients. As maternal anemia during pregnancy has been directly correlated with increased frequency of infant mortality, preterm labor, and unsuccessful pregnancy [21, 28], anemia should be managed aggressively. Erythropoietin has been shown to be safe to pregnant dialysis patients without recognizable increased risks of teratogenicity or hypertension secondary to blood volume overload [15, 17]. Erythropoietin may be increased by 50%–100% in an attempt to achieve targeted hemoglobin levels above 10-11 g/dL, with hematocrit concentrations above 30%–35% [1, 12, 17, 18] and transferrin saturations above 30% [12]. Grossman and colleagues [29] suggested an immediate intravenous administration of 500 mg of iron in all pregnant dialysis patients with transferrin saturation levels below 30%. Maternal complications of thrombosis of arteriovenous fistulas secondary to pregnancy-induced hypercoagulability can be managed safely with heparin [30]. Despite the fact that intravenous iron [6] and heparin [15] seem to be harmless during pregnancy, close observation of iron stores and minimization of heparin doses are highly advised [6, 15].

Malnutrition is frequent among pregnant dialysis patients. The nutritional problems associated with chronic renal insufficiency are further exacerbated by the increased nutritional requirements of pregnancy [31]. It is recommended for pregnant dialysis patients to maintain calorie intake of 30–35 kcal/day [6] and protein intake of 1.8 g/kg/day [2, 6] plus an extra 20 g of protein per day for fetal development in some studies [4]. Folic acid supplements (0.8–1–5 mg/day) should be offered early in the pregnancy (first trimester) to ensure normal neural development [6, 19]. Supplements of water-soluble vitamins should be provided as requirements of such vitamins are largely increased during pregnancy and their elimination is markedly increased through dialysis [4]. Furthermore, it is advised to consume 1500 mg/day of calcium [5]. Human placenta has been shown to be a valuable source of 1,25-dihydroxyvitamin D3 [32]. Vitamin D3 levels should be checked regularly and supplemented only when levels are low [4]. Appropriate correction of all nutritional deficits is essential for reduced fetal mortality and successful pregnancy outcomes.

With respect to obstetric management [4], it is recommended to follow up pregnant dialysis patients and their prospective newborns in the high-risk clinics (units) during the antenatal, perinatal, and postpartum (postnatal) periods. Furthermore, it is advised to frequently monitor maternal blood pressure and amniotic fluid closely (before, during, and after dialysis) [33]. Fetal heart rate should be closely monitored as soon as fetal viability is detected [4]. Fetal nonstress test twice daily and Doppler velocimetry measurements (umbilical artery and middle cerebral artery) once daily are highly recommended [34]. Betamethasone (12 mg, 24 hours apart, two doses) or dexamethasone (6 mg, 12 hours apart, 4 doses) prior to delivery and surfactant therapy after delivery are recommended to promote lung maturation.

Cesarean section appears to be the most frequent mode of delivery among pregnant dialysis patients and is usually driven by obstetric complications such as premature rupture of membrane, polyhydramnios, and preeclampsia [35]. Serial obstetric ultrasound examinations should be conducted on closely regular basis to provide the most appropriate care possible for mother and newborn [4]. Gynecologists should be involved whenever needed.

Lastly, counseling parents about fertility and contraception issues as well as maternal-fetal outcomes in dialysis patients must be discussed extensively so parents can opt for their best decisions.

## References

[1] Holley JL, Reddy SS (2003). Pregnancy in dialysis patients: a review of outcomes, complications, and management. *Seminars in Dialysis*.

[2] Hou SH (1994). Frequency and outcome of pregnancy in women on dialysis. *American Journal of Kidney Diseases*.

[3] Schmidt RJ, Holley JL (1998). Fertility and contraception in end-stage renal disease. *Advances in Renal Replacement Therapy*.

[4] Giatras I, Levy DP, Malone FD, Carlson JA, Jungers P (1998). Pregnancy during dialysis: case report and management guidelines. *Nephrology Dialysis Transplantation*.

[5] Reddy SS, Holley JL (2007). Management of the pregnant chronic dialysis patient. *Advances in Chronic Kidney Disease*.

[6] Hou S (1999). Pregnancy in chronic renal insufficiency and end-stage renal disease. *American Journal of Kidney Diseases*.

[7] Bahadi A, El Kabbaj D, Guelzim K (2010). Pregnancy during hemodialysis: a single center experience. *Saudi Journal of Kidney Diseases and Transplantation*.

[8] Piccoli GB, Conijn A, Consiglio V (2010). Pregnancy in dialysis patients: is the evidence strong enough to lead us to change our counseling policy?. *Clinical Journal of the American Society of Nephrology*.

[9] Bagon JA, Vernaeve H, de Muylder X, Lafontaine J-J, Martens J, van Roost G (1998). Pregnancy and dialysis. *American Journal of Kidney Diseases*.

[10] Okundaye I, Abrinko P, Hou S (1998). Registry of pregnancy in dialysis patients. *American Journal of Kidney Diseases*.

[11] Furaz-Czerpak KR, Fernández-Juárez G, Moreno-de la Higuera MÁ, Corchete-Prats E, Puente-García A, Martín-Hernández R (2012). Pregnancy in women on chronic dialysis: a review. *Nefrologia*.

[12] Jungers P, Chauveau D (1997). Pregnancy in renal disease. *Kidney International*.

[13] Schaefer RM, Kokot F, Wernze H, Geiger H, Heidland A (1989). Improved sexual function in hemodialysis patients on recombinant erythropoietin: a possible role for prolactin. *Clinical Nephrology*.

[14] Confortini P, Galanti G, Ancona G, Giongio A, Bruschi E, Lorenzini E (1971). Full term pregnancy and successful delivery in a patient on chronic hemodialysis. *Proceedings of the European Dialysis and Transplant Association*.

[15] Walsh A-M (2002). Management of a pregnant woman dependent on haemodialysis. *EDTNA-ERCA Journal*.

[16] Hou S, Firanek C (1998). Management of the pregnant dialysis patient. *Advances in Renal Replacement Therapy*.

[17] Vázquez-Rodríguez JG (2010). Hemodialisis and pregnancy: technical aspects. *Cirugia y Cirujanos*.

[18] Hou S (2003). Pregnancy in dialysis patients: where do we go from here?. *Seminars in Dialysis*.

[19] Haase M, Morgera S, Bamberg C (2005). A systematic approach to managing pregnant dialysis patients—the importance of an intensified haemodiafiltration protocol. *Nephrology Dialysis Transplantation*.

[20] Hou S (2002). Modification of dialysis regimens for pregnancy. *International Journal of Artificial Organs*.

[21] Asamiya Y, Otsubo S, Matsuda Y (2009). The importance of low blood urea nitrogen levels in pregnant patients undergoing hemodialysis to optimize birth weight and gestational age. *Kidney International*.

[22] Parker TF, Wingard RL, Husni L, Ikizler TA, Parker RA, Hakim RM (1996). Effect of the membrane biocompatibility on nutritional parameters in chronic hemodialysis patients. *Kidney International*.

[23] Le Curieux F, Marzin D, Erb F (1993). Comparison of three short-term assays: results on seven chemicals. Potential contribution to the control of water genotoxicity. *Mutation Research*.

[24] Hussain SA, Savin V, Piering W, Tomasi J, Blumenthal S (2005). Phosphorus-enriched hemodialysis during pregnancy: two case reports. *Hemodialysis International*.

[25] Chao A-S, Huang J-Y, Lien R, Kung F-T, Chen P-J, Hsieh PCC (2002). Pregnancy in women who undergo long-term hemodialysis. *American Journal of Obstetrics and Gynecology*.

[26] Eroğlu D, Lembet A, Ozdemir FN, Ergin T, Kazanci F, Kuşcu E (2004). Preg­nancy during hemodialysis: perinatal outcome in our cases. *Transplantation Proceedings*.

[27] Paller MS (1998). Hypertension in pregnancy. *Journal of the American Society of Nephrology*.

[28] Levy A, Fraser D, Katz M, Mazor M, Sheiner E (2005). Maternal anemia during pregnancy is an independent risk factor for low birthweight and preterm delivery. *European Journal of Obstetrics Gynecology and Reproductive Biology*.

[29] Grossman SD, Hou S, Moretti MI, Saran S (1993). Nutrition in the pregnant dialysis patient. *Journal of Renal Nutrition*.

[30] Tan L-K, Kanagalingam D, Tan H-K, Choong H-L (2006). Obstetric outcomes in women with end-stage renal failure requiring renal dialysis. *International Journal of Gynecology and Obstetrics*.

[31] Ikizler TA, Hakim RM (1996). Nutrition in end-stage renal disease. *Kidney International*.

[32] Zerwekh JE, Breslau NA (1986). Human placental production of 1*α*,25-dihydroxyvitamin D3: biochemical characterization and production in normal subjects and patients with pseudohypoparathyroidism. *Journal of Clinical Endocrinology and Metabolism*.

[33] Brost BC, Newman RB, Fries M, Calhoun BC (1996). The effects of hemodialysis on total intrauterine volume. *Ultrasound in Obstetrics and Gynecology*.

[34] Oosterhof H, Navis GJ, Go JG, Dassel ACM, de Jong PE, Aarnoudse JG (1993). Pregnancy in a patient on chronic haemodialysis: fetal monitoring by Doppler velocimetry of the umbilical artery. *British Journal of Obstetrics and Gynaecology*.

[35] Al-Saran KA, Sabry AA (2008). Pregnancy in dialysis patients: a case series. *Journal of Medical Case Reports*.

